# Different Research Approaches in Unraveling the Venom Proteome of *Naja ashei*

**DOI:** 10.3390/biom10091282

**Published:** 2020-09-05

**Authors:** Konrad Kamil Hus, Łukasz Marczak, Vladimír Petrilla, Monika Petrillová, Jaroslav Legáth, Aleksandra Bocian

**Affiliations:** 1 Department of Biotechnology and Bioinformatics, Faculty of Chemistry, Rzeszow University of Technology, Powstańców Warszawy 6, 35-959 Rzeszów, Poland; knr.hus@gmail.com (K.K.H.); Jaroslav.Legath@uvlf.sk (J.L.); 2 Institute of Bioorganic Chemistry, Polish Academy of Sciences, Noskowskiego 12/14, 61-704 Poznań, Poland; lukasmar@ibch.poznan.pl; 3 Department of Physiology, University of Veterinary Medicine and Pharmacy, Komenského 73, 041 81 Košice, Slovakia; petrillav@gmail.com; 4 Zoological Department, Zoological Garden Košice, Široká 31, 040 06 Košice-Kavečany, Slovakia; 5 Department of General Education Subjects, University of Veterinary Medicine and Pharmacy, Komenského 73, 041 81 Kosice, Slovakia; monika.petrillova@uvlf.sk; 6 Department of Pharmacology and Toxicology, University of Veterinary Medicine and Pharmacy, Komenského 73, 041 81 Košice, Slovakia

**Keywords:** snake venom, venomics, absolute protein quantification, label-free shotgun mass spectrometry

## Abstract

The dynamic development of venomics in recent years has resulted in a significant increase in publicly available proteomic data. The information contained therein is often used for comparisons between different datasets and to draw biological conclusions therefrom. In this article, we aimed to show the possible differences that can arise, in the final results of the proteomic experiment, while using different research workflows. We applied two software solutions (PeptideShaker and MaxQuant) to process data from shotgun LC-MS/MS analysis of *Naja ashei* venom and collate it with the previous report concerning this species. We were able to provide new information regarding the protein composition of this venom but also present the qualitative and quantitative limitations of currently used proteomic methods. Moreover, we reported a rapid and straightforward technique for the separation of the fraction of proteins from the three-finger toxin family. Our results underline the necessary caution in the interpretation of data based on a comparative analysis of data derived from different studies.

## 1. Introduction

Over the past decades, we have witnessed continuous progress in venom research, which has resulted in the gathering of a substantial amount of invaluable data on the biochemical composition of many venoms, the mechanism of their action, or their potential use in medicine to design drugs or improve current therapies to treat snakebite envenomation. The emergence of new research concepts, technological advances as well as the implementation of appropriate bioinformatics platforms, are the factors that made it possible to solve many of the problems that venomics have been struggling with for years. However, even with such great scientific progress, there is still no single technique capable to unambiguously assign protein identity to every venom component [1,2]. Issues related to the presence of shared peptides, the problem with missing data due to the high dynamic range of venoms and paucity of comprehensive protein databases are the hindrances that currently impede the development of peptide-centric methods [3,4,5]. On the other hand, top-down approaches are still in the early stages of development and it seems that we still have to wait until MS analysis of intact proteins have the chance to become a method of choice in venom studies [1].

On top of that, despite the plethora of different approaches for absolute quantification of venom proteins, none of them can be recognized as impeccable. In this manner, label-based techniques suffer from the lack of proper standards that could accurately reflect the complexity of venom mixtures, while the current algorithms used in label-free methods are at best a good approximation of the real picture [1,5,6,7].

For these reasons, to estimate venom proteome with sufficient fidelity, we are forced to apply a sophisticated research workflow consisting of many individual techniques, each providing unique information about the sample. The ideal example of such an approach is the snake venomics protocol, firstly introduced in 2004 by Calvete’s team, which is still considered as the gold standard in proteomic research of venoms. This workflow, however, consists of cysteine mapping, N-terminal sequencing, and MS analysis of fractions separated by RP-HPLC and SDS-PAGE respectively, is very time-consuming and requires an application of techniques that are not routinely used in every laboratory [8]. Therefore, very often, different research groups decide to rely on methods that are faster and available to them. Application of different techniques for the preparation, decomplexation, and measurement of the venom samples leads to the fact that the data obtained by different research teams may significantly differ. If we then add several various procedures for further data processing, based on distinct concepts and algorithms, it is extremely difficult to draw accurate and reliable conclusions from comparing proteomic data between different studies [9].

In 2018, we presented the first data regarding the proteome of *Naja ashei* venom with the use of two-dimensional electrophoresis coupled with MALDI-ToF spectrometer. We were able to discover 7 protein families, of which three-finger toxins (3FTxs) and phospholipases A_2_ (PLA_2_) constituted the vast majority [10]. This report supplements qualitative information for this venom but also collates the results obtained using different analytical methods and data processing protocols. We compared previous data for *Naja ashei* venom proteome obtained by 2DE-MS strategy with the data acquired by label-free shotgun mass spectrometry (for crude and fractionated venom), and processed by two different software for proteomic data analysis—PeptideShaker and MaxQuant.

## 2. Materials and Methods

### 2.1. Sample Pre-Fractionation and Preparation for LC-MS/MS Analysis

A pooled sample of *Naja ashei* venom was obtained from two adult snakes (male and female) captured in Kenya. The same sample was used in our previous proteomic analysis [10]. After extraction, venom was stored at −20 °C (transport temperature) and then moved to −80 °C for deep freezing. Venom milking took place in the breeding garden Pata near Hlohovec (Slovakia), which had been designed for reptiles´ conservation of the gene pool under the veterinary certificate No. CHEZ-TT-01. The breeding garden also serves as a quarantine station for imported animals and is an official importer of exotic animals from around the world, having the permission of the State Nature Protection of the Slovak Republic under the No. 03418/06, the trade with endangered species of wild fauna and flora and on amendments to certain laws under Law no. 237/2002 Z.z.

Crude venom was diluted at a 1:80 and 1:100 (*v*/*v*) ratios with 50 mM ammonium bicarbonate pH 8. 1:80 (*v*/*v*) aliquot was directly taken to further analysis as a dilution of crude venom, while a 100-fold dilution sample was subjected to the additional step of pre-fractionation. It was centrifuged for 8 min at 12,400× *g* using 0.5 mL Microcon^®^-30 kDa centrifugal filter unit with Ultracel^®^-30 membrane (Merck Millipore, Cork, Ireland). The bottom fraction was transferred to the new test tube, while the upper portion was moved to the new centrifugal unit, placed upside down, and spun for 3 min at 1000× *g*. Approximately three-quarters of the initial sample volume passed through the membrane, while a quarter remained in the upper fraction. Protein concentration in both types of samples (fractionated and non-fractionated) was measured using Pierce^™^ BCA Protein Assay Kit (Thermo Fisher Scientific, Waltham, MA, USA). For digestion, samples were prepared by mixing volumes containing 4.3 μg of proteins with 50 mM ammonium bicarbonate pH 8, to a final volume of 25 μL. Proteins were reduced with DTT (final concentration: 7 mM) for 5 min at 95 °C and then alkylated with IAA (final concentration: 10 mM) for 20 min in the dark. The samples were digested with 0.2 μg trypsin (Promega, Madison, WI, USA) for 18 h at 37 °C.

### 2.2. SDS-PAGE of Fractions Obtained after Membrane Filtration

Fractions obtained after centrifugation on 30 kDa membranes were subjected to SDS-PAGE analysis under reducing and non-reducing conditions. Samples were mixed with sample buffer (62.5 mM Tris-HCl pH 6.8, 2% SDS, 10% glycerol, 0.002% bromophenol blue with or without 5% β-mercaptoethanol) in 1:4 ratio (*v*/*v*) and were heated at 95 °C for 5 min. Then, 15 μL of each sample and two protein mass markers (ROTI^®^Mark BI-PINK and ROTI^®^Mark TRICOLOR; Roth, Karlsruhe, Germany) were loaded onto the 17% (with 5% stacking gels) acrylamide gels. SDS-PAGE was performed using the Mini-Protean II apparatus (Bio-Rad Laboratories, Inc., Hercules, CA, USA). Gels were stained overnight with colloidal Coomassie Brilliant Blue G-250. To ensure the reproducibility of the results, the SDS-PAGE experiment was performed with fractionated samples obtained with filters deriving from two distinct batches of product.

### 2.3. Shotgun LC-MS/MS Analysis

Firstly, 0.86 μg of digested peptides from each sample was taken for LC-MS/MS analysis. Peptide separation was performed on a Dionex Ultimate 3000 RSLC NanoLC system (Thermo Fisher Scientific, Waltham, MA, USA) using Acclaim PepMap RSLC nanoViper C18 column (75 μm × 25 cm; 2 μm granulation) (Thermo Fisher Scientific, Waltham, MA, USA) with 180 min ACN gradient (from 4% to 60%; in 0.1% formic acid). Ion signals were detected on Q Exactive Orbitrap mass spectrometer (Thermo Scientific, Waltham, MA, USA) operating in on-line mode with LC system. The analysis was conducted in data-dependent acquisition (DDA) mode with survey scans acquired at a resolution of 70,000 at *m*/*z* 200 in MS mode, and 17,500 at *m*/*z* 200 in MS2 mode. Spectra were recorded in the scanning range of 300–2000 *m*/*z* in positive ion mode. Higher energy collisional dissociation (HCD) ion fragmentation was performed with normalized collision energies set to 25.

### 2.4. MS Data Processing Protocol

#### 2.4.1. Qualitative and Quantitative Analysis in MaxQuant

The acquired MS/MS raw data files were analyzed using MaxQuant software (ver. 1.6.7.0). Protein identification was conducted according to UniProtKB Serpentes database (release 9/2019) using Andromeda engine. Carbamidomethylation (C) was set as fixed modification while oxidation (M) and acetyl (protein N-term) was used as variable modifications. Mass tolerance was set to 20 ppm for initial MS search, 4.5 ppm for main MS search and 20 ppm for MS/MS fragment ions. Trypsin with full specificity and maximum two missed cleavages was applied for enzyme properties. PSM and protein False Discovery Rate (FDR) was set to 1%. Hits that were identified only by site, found in decoy or contaminant lists, and were identified with less than 2 peptides, were subsequently filtered out. At this step, all hits were manually revised for proteins that were unlikely to be the components of snake venoms and filtered out. iBAQ (intensity-based absolute quantification) values of razor and unique peptides were used for the calculation of the amount of particular protein in the sample. Then, the proteins were assigned to different protein groups and the percentage of each protein group was calculated by dividing the summed iBAQ values of proteins assigned to the group by the summed iBAQ values of all quantified proteins identified in the sample.

#### 2.4.2. Qualitative and Quantitative Analysis in PeptideShaker

Peak lists obtained from MS/MS spectra were identified using X! Tandem Vengeance (ver. 2015.12.15.2) and MS-GF+ (ver. 2018.04.09) search engines using SearchGUI (ver. 3.3.16). Protein identification was conducted against a concatenated target/decoy (reversed) UniProtKB Serpentes database (release 9/2019; 150253 target sequences). The identification settings were as follows: Trypsin (Semi-Specific), with a maximum of 2 missed cleavages; 10.0 ppm as MS1 and 0.02 Da as MS2 tolerances; fixed modifications: Carbamidomethylation of C, variable modifications: Oxidation of M, Acetylation of protein N-term, fixed modifications during refinement procedure: Carbamidomethylation of C, variable modifications during refinement procedure: Pyrolidone from E, Pyrolidone from Q, Pyrolidone from carbamidomethylated C.

Peptides and proteins were inferred from the spectrum identification results using PeptideShaker version 1.16.42. Peptide Spectrum Matches (PSMs), peptides and proteins were validated at a 1.0% False Discovery Rate (FDR) estimated using the decoy hit distribution. Moreover, all hits were manually revised for proteins that were unlikely to be the components of snake venoms and filtered out.

Spectrum counting abundance indexes were estimated using the Normalized Spectrum Abundance Factor (NSAF+ algorithm). Identified proteins were manually assigned to different families and final quantitative values for the whole family were calculated by summing all individual NSAF+ values of proteins assigned to certain family divided by the sum of NSAF+ of all identified proteins.

The mass spectrometry proteomics data have been deposited to the ProteomeXchange Consortium via the PRIDE partner repository with the dataset identifier PXD020924 and 10.6019/ PXD020924.

## 3. Results

### 3.1. Comparative Analysis of the Results for the Naja ashei Venom Proteome Obtained Using Different Research Workflows

**Figure 1:**
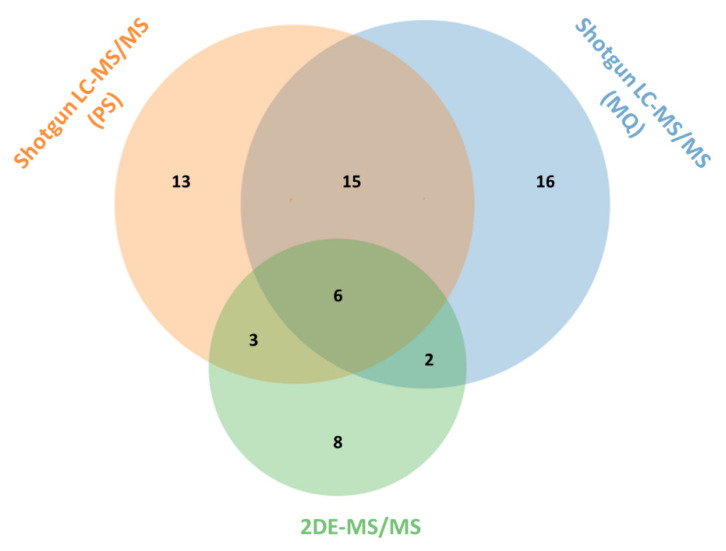
The comparison of the number of proteins identified using different research workflows. Shotgun LC-MS/MS strategy yielded in the identification of 37 and 39 proteins for PeptideShaker (PS) and MaxQuant (MQ) approaches, respectively. Both software provided a relatively high share of workflow-specific proteins. Previous 2DE-MS/MS analysis allowed for the detection of 19 proteins.

In our previous 2DE-MS analysis of *Naja ashei* venom proteome, we were able to identify 19 proteins that belong to 7 different protein families [10]. Here, we applied two different data processing workflows to analyze data acquired by shotgun LC-MS/MS proteomic experiment. Raw data was used as an input for either PeptideShaker (PS) or MaxQuant (MQ) software to identify and quantify proteins in venom. PeptideShaker, which applied X! Tandem and MS-GF+ search engines, was able to identify 37 proteins, while MaxQuant, operating with Andromeda engine, yielded 39 protein hits. Cross-comparison of the number of proteins identified with the use of three different research workflows is presented in Figure 1.

With the highest number of overall protein identifications, MaxQuant also provided the greatest number of unique hits (16 proteins). In this manner, PS software contributed 13 unique identifications, while 2DE-MS/MS strategy allowed for the detection of only 8 exclusive hits. However, it should be noted that in our previous proteomic approach, Mascot search was applied only against the Swiss-Prot repository, while the database for shotgun proteomics was prepared from curated and non-curated sections of UniProtKB. In fact, a large proportion of these unique proteins, reported by the search engines in both research workflows (PS and MQ), were identified in the TrEMBL database.

Nevertheless, although 21 proteins were present in the results of both MQ and PS approaches, the high number of workflow-specific hits is also distinctive. In this case, however, we have not observed any tendency towards the detectability of certain groups of proteins using any particular research workflow (Tables S1 and S2).

In the context of this comparison, it should be emphasized that the “protein inference problem”, which is an inherent part of the bottom-up strategy, makes drawing conclusions based on individual proteins, more an approximation rather than certainty. Hence, it would be more reliable to compare whole protein families that were detected with different approaches. Table 1 shows that MaxQuant was able to detect proteins belonging to 13 protein families. These were two more families than in the analysis with PeptideShaker, and six more than in the 2DE-MS approach. This difference, however, mainly concerned low-copy protein families, whose share in the venom did not exceed 1%. Moreover, such a small number of detected protein families in the case of the 2DE-MS/MS strategy was, for certain, partly due to the database, as mentioned above, but also partly due to the insufficient resolution that was observable in previous 2D protein maps.

**Figure 2:**
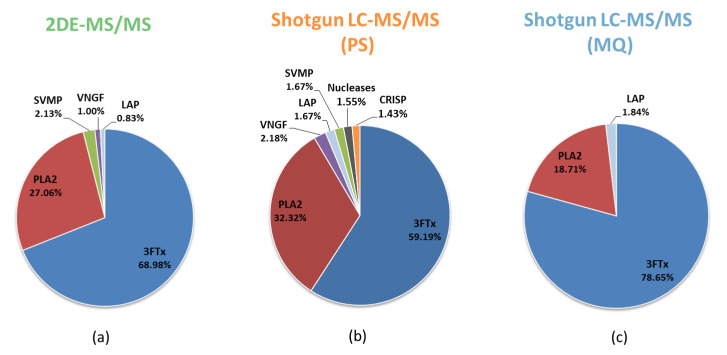
The percentage distribution of different protein families in *Naja ashei* venom proteome calculated on the basis of different research workflows: (**a**) densitometry after 2DE-MS/MS analysis; (**b**) Shotgun LC-MS/MS proteomics processed by PeptideShaker; (**c**) Shotgun LC-MS/MS proteomics processed by MaxQuant; LAP (Low abundant proteins)—proteins whose total percentage share did not exceed 1% were included in the LAP group. The complete list of proteins with its quantitative data is provided in Table S1.

However, the most notable differences in the results of distinct research workflows can be seen in the quantification of the venom proteins. In the publication from 2018, we quantified the proportions of individual protein families in venom by densitometry of Coomassie-stained 2D gels. We reported that the composition of venom is dominated by two protein families, which together constitute more than 95% of venom (68.98% 3FTx, 27.06% PLA_2_). Now, we collate this data with the information obtained from the shotgun LC-MS/MS experiment but processed with two different software solutions. Although we confirmed the prevalence of three-finger toxins and phospholipases A_2_, the comparison of the accurate values clearly shows that the applied methodology has a very significant influence on the final quantitative results (Figure 2).

The quantitative differences are most pronounced in the dominant protein families and can vary by 19.46% for three-finger toxins or 13.61% for phospholipases A_2_. Moreover, if we assume the authenticity of any of these datasets, we may almost double over- or underestimate the true percentage of PLA_2_ in the case of the wrong method selection. It is worth mentioning that these differences exist between the data that differed only in the protocol of data processing.

Considering that the label-free absolute quantification protocol in both programs is carried out using completely different algorithms (NSAF+ in PeptideShaker; iBAQ in MaxQuant), the occurrence of differences in the results is not particularly surprising. Nonetheless, comparing absolute quantitative data between studies that used different analytical workflows, but also different data processing protocols, can be burdened with a high risk of error.

### 3.2. LC-MS/MS Analysis of Naja ashei Venom after Sample Decomplexation with the Use of Different Data Processing Software and an SDS-PAGE of Obtained Fractions

**Figure 3:**
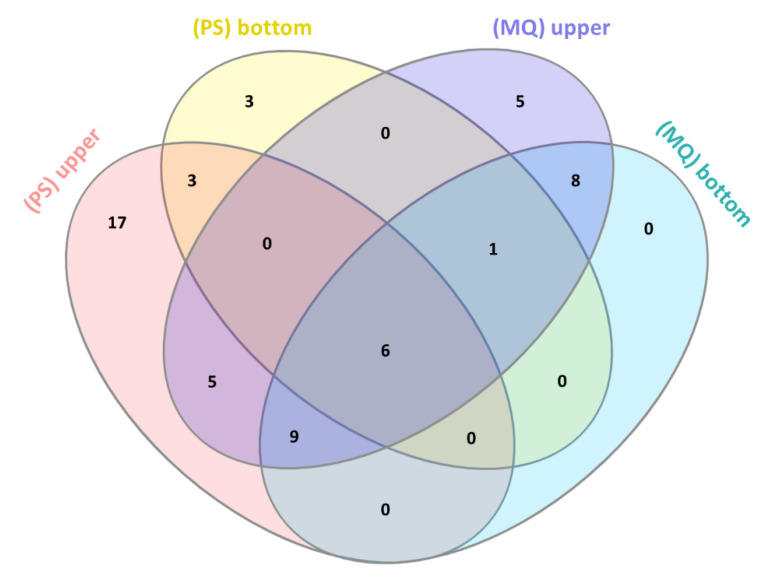
The comparison of the number of proteins identified in the upper and the bottom fractions using PeptideShaker (PS) and MaxQuant (MQ) software. In overall, PeptideShaker identified 40 proteins in the upper and 13 proteins in the bottom fraction, while MaxQuant reported 34 hits for the upper and 24 hits for the bottom fraction.

To reduce the complexity of the venom sample and increase the number of identifications, we applied a simple step of fractionation using 30 kDa centrifuge filters. As a result, we obtained two fractions with protein concentrations of 11.792 μg/μL in the upper fraction and 0.430 μg/μL in the bottom fraction. The qualitative results of the LC-MS/MS analysis are presented in Figure 3 and Table 2.

As might be expected, the decomplexation of the sample allowed for the total increase in the number of identifications in both data processing protocols, compared to the analysis of unfractionated (crude) venom. Again, the total number of identified proteins in both fractions was higher for MQ (58 proteins) than for PS (53 proteins). It was mainly influenced by the considerable difference in the number of hits identified in the bottom fraction. MaxQuant identified fewer proteins (34) in the upper fraction but reported 24 identifications for the bottom fraction. On the other hand, PS identified 40 proteins in the upper fraction but only 13 in the bottom one. This time, however, the highest number of unique hits was delivered by the combination of X! Tandem and MS-GF+ search engines and was equal to 17 proteins. Six proteins were included in all results, while there were no unique hits in the MaxQuant output for the bottom fraction. Again, no clear trends were observed in the identification of specific groups of proteins by different software (Table S2).

At this point, it is worth noting that after filtration, we observed an overall increase in the number of unique protein hits (previously unidentified in the crude sample) by 16 for the PS analysis and by only 5 proteins for the MQ analysis. On the other hand, the number of proteins identified only in the unfiltered sample was 9 and 10, respectively for the PS and MQ analysis. Thus, from this perspective, the combination of two search engines in the case of PS analysis provided more diversified results for filtrated samples (Table S2).

Interestingly, although the decomplexation of the sample resulted in an overall increase in the number of identified proteins, it did not necessarily translate into a greater number of recorded peptides. This is particularly visible in the case of MQ analysis, where the amount of unique peptides is significantly higher in the unfiltered sample (216 peptides) compared to the sum of non-redundant peptides in samples after fractionation (154 peptides). This deceptive contradiction, however, can be explained, as the proteins detected in the unfiltered sample were on average identified from a larger number of peptides (Table S3). Moreover, as was mentioned before, the higher variability of identified proteins in MQ analysis appeared in the unfiltered sample.

Nevertheless, after sample decomplexation, we have also observed an increase in the total number of identified protein families from 11 to 13 in the case of PeptideShaker. Moreover, only in this case, it was possible to detect TF-like protein (UniProt ID - A0A2H6N0F2), which was not earlier achievable under any other conditions (Table 2).

Furthermore, we were able to observe the prevalence of low-molecular-weight proteins in the results of the bottom fraction, in the case of data processed by both software solutions. This is not particularly surprising as we used the 30 kDa filter membrane, but in the list of identified proteins for this fraction, larger proteins were observed as well. It might be the case that they were included in the bottom fraction due to the partial degradation of some proteins in the sample.

In this context, a more confusing thing is the dominance of many low-molecular-weight proteins in the upper fraction. This, however, could be explained by an incomplete process of filtration or unspecific membrane-protein interaction ending with the adhesion of proteins to the membrane. A possible explanation for this phenomenon may also be a high concentration of the fractionated sample, resulting in the clogging of the membrane pores.

**Figure 4:**
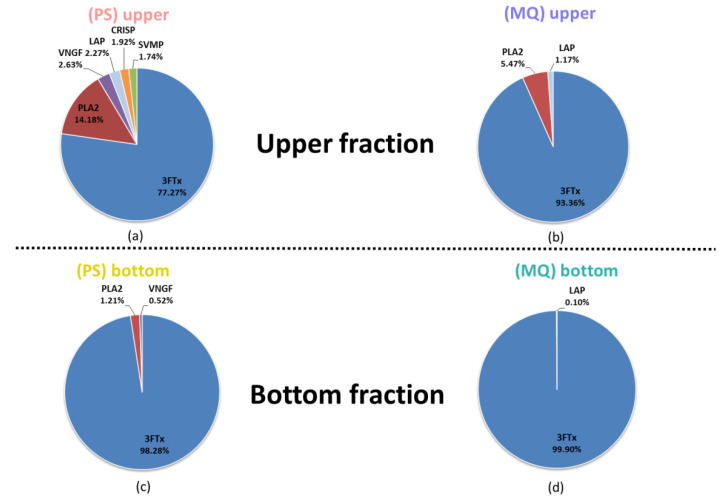
The percentage distribution of different protein families in upper fraction: (**a**) calculated with PeptideShaker (PS); (**b**) calculated by MaxQuant (MQ); and bottom fraction: (**c**) calculated with PeptideShaker; (**d**) calculated by MaxQuant (MQ). LAP (Low abundant proteins)—proteins whose total percentage share did not exceed 1% were included in the LAP group.

Another unexpected issue to consider is represented by the quantitative results of the shotgun LC-MS/MS proteomic experiment, after sample fractionation (Figure 4).

Originally, we expected to see a higher proportion of low abundant proteins in the upper venom fraction after the initial sample decomplexation. That was partly because two dominant protein families in *Naja ashei* venom have a lower molecular mass than the mass cut-off of membranes used in the experiment. While, in fact, in the bottom fraction we observed almost exclusively proteins from the three-finger toxin family, quite surprisingly, in the upper fraction, the percentage share for this group of proteins also increased. In comparison to the crude venom analysis, this increase was very significant, as in the case of the PeptideShaker calculations it equaled 18.08%, and in the case of the MaxQuant, 14.71%. This observation seems unusual as after filtering some of the 3FTx proteins into the bottom fraction, we would expect a decrease in their percentage in the upper fraction compared to their amount in the whole venom. Based on these results, it could be assumed that the real amount of proteins from this family in this venom is much higher than initially reported. It cannot be ruled out that the stochastic nature of data-dependent acquisition in this case largely influences the data particularly concerning this family of proteins. In other words, it is possible that during MS analysis, part of the 3FTx proteins mask the presence of other proteins from this family, which are also present there in a considerable amount.

**Figure 5:**
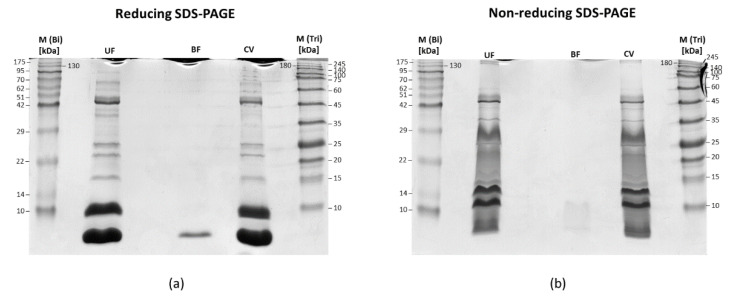
Representative gel images of the samples separated with SDS-PAGE under (**a**) reducing and (**b**) non-reducing conditions. Electrophoresis was performed on 17% stacking gels. UF (Upper fraction); BF (Bottom fraction); CV (Crude venom); M(Bi) (ROTI^®^Mark BI-PINK protein mass marker); M(Tri) (ROTI^®^Mark TRICOLOR mass marker).

It is difficult to neglect that the bottom fraction almost exclusively consists of proteins from the 3FTx family. This could mean that a simple method based on the use of centrifuge filters can be a very rapid and efficient way to isolate from venom a certain share of three-finger toxin proteins. However, to confirm these results, we have performed an SDS-PAGE analysis of the obtained fractions under reducing and non-reducing conditions (Figure 5).

The obtained gels confirm the results acquired from the LC-MS/MS experiment. The upper fraction largely reflects the composition of whole unfractionated venom, while the bottom fraction consists almost entirely of proteins with a mass of about 7 kDa. Electrophoresis under non-reducing conditions revealed that these proteins form a kind of larger aggregates with a mass close to 11 kDa. At the same time, the non-reducing electrophoresis ruled out the hypothesis that the low-molecular-weight proteins in the upper fraction formed large multimers, which resulted in their mass shift above 30 kDa. It is easy to notice that even in non-reducing conditions, the upper fraction is still mostly composed of low-molecular-weight proteins below 30 kDa. Moreover, SDS-PAGE electrophoresis confirmed that, with the use of centrifuge filters, a separation, of a certain fraction of 3FTx proteins from *Naja ashei* venom, is possible. Likely, this method may also be effective with other venoms of similar composition.

## 4. Discussion

The field of venomics has recently experienced a tremendous boost, especially in the context of novel concepts implemented to the instrumentation of high-throughput analytical techniques as well as in data analysis workflows. Nevertheless, there are still many issues limiting the scientific capacity of modern proteomic methods, which can be broadly classified into qualitative- and quantitative-related. One of the problems connected with qualitative analysis in data-dependent mode is the suppression of less numerous peptides by the high abundant ones. In DDA mode, this is partly resolved by rapid filtration of the precursor ions that have already been analyzed, allowing the selection of other peptides. In many situations, this solution can be very beneficial as it aims to increase the coverage and heterogeneity of the detected ion population. However, at the same time, it excludes from the analysis the co-eluting precursors of similar mass [11]. Therefore, in the case of samples containing peptides with a similar sequence and the resulting physicochemical properties, this effect can have a significant impact on the final results. We suspect that we were able to observe such an effect, in the case of proteins from the same, 3FTx family. It is possible, that a large proportion of 3FTx precursors was ignored during the MS analysis of crude venom, and their presence was revealed only after the sample decomplexation. This could be an explanation of the unexpected increase in the proportion of three-finger toxins in the upper fraction compared to the unfractionated venom.

On the other hand, the major bottleneck of label-free absolute protein quantification remains in the insufficient accuracy of available algorithms. They operate under the assumption that there is a linear relationship between protein abundance and measured MS-based parameters (like the ion intensity or the number of recorded spectra). These algorithms apply different strategies to tackle the common limitations of mass spectrometry resulting from different ion ionizability, or the problem with missing values, however, they are still not capable of accounting for all the drawbacks of quantitative proteomics [12,13,14]. In this study, for quantitative analysis, we applied the NSAF+ algorithm implemented in PeptideShaker and riBAQ (relative iBAQ) values that are available in MaxQuant. NSAF+ is an algorithm based on spectral counting, which also takes into consideration the length of analyzed proteins as well as shared and redundant peptides [15]. However, solutions that rely on quantifying the number of spectral events for certain peptides and proteins are still considered as a rough estimate [14,16,17]. The iBAQ parameter is calculated as the sum of the intensities of all precursor ions assigned to a given protein divided by the number of all its theoretically observable peptides. riBAQ is additionally normalized by dividing the iBAQ parameter for a certain protein, by the sum of all iBAQ values derived from other identified proteins [6,18]. Although the iBAQ parameter repeatedly showed sufficient accuracy in estimating the absolute content of different proteins [13,16], it has been previously reported that this algorithm tends to significantly underestimate the actual amount of low abundant proteins [12]. This could partially explain the observed, highest percentage values of the 3FTx family, reported in every case by MaxQuant.

As it was presented, the applied algorithms can greatly influence the final results, making any data comparisons very difficult. In this context, it seems that the venomics protocol for quantitative analysis, although laborious, remains the most accurate method available. Although the methods proposed therein are not without the limitations of quantitative methods, especially in the context of biases related to the presence of certain amino acids in protein sequences [19], the three-leveled quantification workflow still seems the best available solution [1].

At the moment, unfortunately, the problem of precise quantitative analysis in venom studies still seems unsolved. Nevertheless, the first positive experiences with elemental mass spectrometry (ICP-MS) offer an opportunity to solve these problems in the future. To make it possible, however, it will be necessary to develop and configure the currently used apparatus systems [1,5].

In comparison to the original 2DE-MS proteomic analysis, in this study, we were able to provide information concerning seven new protein families that are also present in *Naja ashei* venom [10]. However, according to the analysis of *Naja ashei* fractions obtained after IEX chromatography, it is known that this venom contains even more low-abundant protein families, undetected in this experiment [20]. This underlines the importance of proper sample prefractionation, in order to obtain the highest possible coverage of analyzed proteome.

Finally, it is worth mentioning that the simple method for 3FTx isolation, presented by us, has the potential to become an initial or targeted separation method for 3FTx proteins. It is possible that by controlling the centrifugation conditions, it would be achievable to obtain fractions with an even higher fraction of three-finger toxins. Probably, this method would also be effective in the case of venoms with similar protein composition.

## 5. Conclusions

Current high-throughput analytical methods produce enormous amounts of experimental data, which then have to be analyzed with the use of sophisticated bioinformatics tools. Therefore, it is important to plan the experiment with full knowledge concerning the pros and cons of the applied analytical techniques as well as tools used for the data processing. In this article, we have shown how the results of the same analysis may differ after using different methods for identification and quantification of proteins. These results also draw attention to the necessary caution in the interpretation of data from such experiments, with particular emphasis on the comparisons of data derived from different studies.

## Figures and Tables

**Table 1:** Protein families detected in *Naja ashei* venom using different research workflows. The values in brackets represent the percentage share of a given group in the proteome, determined using workflow-specific quantitative analysis.

	Research Workflow		
Protein Family	2DE-MS/MS	Shotgun LC-MS/MS (PS)	Shotgun LC-MS/MS (MQ)
3FTx	+(68.98%)	+(59.19%)	+(79.28%)
PLA_2_	+(27.06%)	+(32.32%)	+(18.86%)
SVMP	+(2.13%)	+(2.26%)	+(0.43%)
VNGF	+(1.00%)	+(2.18%)	+(0.81%)
CRISP	+(0.70%)	+(1.43%)	+(0.26%)
CVF	+(0.12%)	+(0.14%)	+(0.02%)
Nucleases	+(0.01%)	+(1.55%)	+(0.10%)
Ig-like	-	+(0.31%)	+(0.08%)
GPx	-	+(0.31%)	+(0.05%)
LAAO	-	+(0.21%)	+(0.02%)
PDE	-	+(0.11%)	+(0.01%)
KUN	-	-	+(0.09%)
PLB	-	-	+(<0.01%)

3FTx (Three-finger toxin); PLA_2_ (Phospholipase A_2_); SVMP (Snake venom metalloproteinase); VNGF (Venom nerve growth factor); CRISP (Cysteine-rich secretory protein); CVF (Cobra venom factor); Ig-like (Immunoglobulin-like protein); GPx (Glutathione peroxidase); LAAO (L-amino acid oxidase); PDE (Phosphodiesterase); KUN (Kunitz-type serine protease inhibitor); PLB (Phospholipase B).

**Table 2:** Protein families detected in fractions of *Naja ashei* venom using PeptideShaker (PS) or MaxQuant (MQ) software. The values in brackets represent the percentage share of a given group in a certain fraction but they are only included if the amount of certain family was not below one-thousandth of a percent. All values are available in Table S1.

	Venom Fraction (Software)			
Protein Family	Upper Fraction (PS)	Bottom Fraction (PS)	Upper Fraction (MQ)	Bottom Fraction (MQ)
3FTx	+(77.27%)	+(98.28%)	+(93.36%)	+(99.90%)
PLA_2_	+(14.18%)	+(1.21%)	+(5.47%)	+(0.08%)
SVMP	+(1.74%)	-	+(0.20%)	+(0.001%)
VNGF	+(2.63%)	+(0.52%)	+(0.54%)	+(0.02%)
CRISP	+(1.92%)	-	+(0.32%)	+(0.005%)
CVF	+(0.12%)	-	+(0.005%)	+(0.001%)
Nucleases	+(0.90%)	-	+(0.03%)	+
Ig-like	+(0.37%)	-	+(0.01%)	+
GPx	+(0.45%)	-	+(0.04%)	+
LAAO	+(0.16%)	-	+(0.02%)	+
PDE	+(0.11%)	-	-	-
KUN	-	-	+(0.007%)	+(0.01%)
PLB	+(0.09%)	-	-	-
TF-like	+(0.07%)	-	-	-

TF-like (Transferrin-like protein).

## Supplementary Materials

The following are available online at https://www.mdpi.com/2218-273X/10/9/1282/s1, Table S1: Complete list of all identified proteins and peptides with its quantitation analysis. Table S2: Cross-comparison of the number of proteins identified with different research workflows. Table S3: Cross-comparison of the number of peptides identified with different research workflows.
